# SARS-CoV-2 Nucleocapsid Plasma Antigen for Diagnosis and Monitoring of COVID-19

**DOI:** 10.1093/clinchem/hvab216

**Published:** 2021-10-04

**Authors:** Hannah Wang, Catherine A Hogan, Michelle Verghese, Daniel Solis, Mamdouh Sibai, ChunHong Huang, Katharina Röltgen, Bryan A Stevens, Fumiko Yamamoto, Malaya K Sahoo, James Zehnder, Scott D Boyd, Benjamin A Pinsky

**Affiliations:** 1 Department of Pathology, Stanford University School of Medicine, Stanford, CA, USA; 2 Division of Infectious Diseases and Geographic Medicine, Department of Medicine, Stanford University School of Medicine, Stanford, CA, USA

## Abstract

**Background:**

Detection of SARS-CoV-2 nucleocapsid antigen in blood has been described, but the diagnostic and prognostic role of antigenemia is not well understood. This study aimed to determine the frequency, duration, and concentration of nucleocapsid antigen in plasma and its association with COVID-19 severity.

**Methods:**

We utilized an ultrasensitive electrochemiluminescence immunoassay targeting SARS-CoV-2 nucleocapsid antigen to evaluate 777 plasma samples from 104 individuals with COVID-19. We compared plasma antigen to respiratory nucleic acid amplification testing (NAAT) in 74 individuals with COVID-19 from samples collected ± 1 day of diagnostic respiratory NAAT, and in 52 SARS-CoV-2-negative individuals. We used Kruskal-Wallis tests, multivariable logistic regression, and mixed-effects modeling to evaluate whether plasma antigen concentration was associated with disease severity.

**Results:**

Plasma antigen had 91.9% (95% CI 83.2-97.0%) clinical sensitivity and 94.2% (84.1-98.8%) clinical specificity. Antigen-negative plasma samples belonged to patients with later respiratory cycle thresholds (C_t_) when compared with antigen-positive plasma samples. Median plasma antigen concentration (log_10_ fg/mL) was 5.4 (IQR 3.9-6.0) in outpatients, 6.0 (5.4-6.5) in inpatients, and 6.6 (6.1-7.2) in intensive care unit (ICU) patients. In models adjusted for age, sex, diabetes, and hypertension, plasma antigen concentration at diagnosis was associated with ICU admission (OR 2.8 [95% CI 1.2-6.2], *P*=.01), but not with non-ICU hospitalization. Rate of antigen decrease was not associated with disease severity.

**Conclusions:**

SARS-CoV-2 plasma nucleocapsid antigen exhibited comparable diagnostic performance to upper respiratory NAAT, especially among those with late respiratory C_t_. In addition to currently available tools, antigenemia may facilitate patient triage to optimize intensive care utilization.

The global response to the coronavirus disease 2019 (COVID-19) pandemic has necessitated the rapid development and widespread deployment of innovative diagnostic tests for the detection of its causative agent, severe acute respiratory syndrome coronavirus-2 (SARS-CoV-2). However, it has been a major challenge to identify prognostic biomarkers to aid in clinical risk stratification.

Identifying those indviduals likely to develop severe COVID-19 is critical for the efficient use of healthcare resources, including the need for intensive care unit (ICU) admission. This risk assessment is particularly important in surge settings where rapid and accurate patient triaging has the potential to reduce morbidity and mortality. It is estimated that 10% of individuals with confirmed SARS-CoV-2 infection develop severe disease (1).

Various routine laboratory tests for host biomarkers, including absolute lymphocyte count, C-reactive protein, ferritin, D-dimer, interleukin-6, procalcitonin, and cardiac troponin have been evaluated for their prognostic utility. Their test performance, however, has proven insufficient for routine clinical decision-making (1–4). Clinical scoring systems, many of which include the above laboratory parameters, have also been investigated, though further validation of the most promising models has been recommended (5).

Additionally, detection and/or quantification of viral components have been considered for their prognostic value. Higher viral burden in upper respiratory specimens was associated with COVID-19 severity in at least one cohort (6), but not others (7,8). Furthermore, a number of studies have demonstrated that the presence of SARS-CoV-2 ribonucleic acid (RNA) in plasma at diagnosis is associated with the development of severe COVID-19 (9–12).

Despite this association between RNAemia and COVID-19 severity, RNA concentrations in plasma are typically near the limit of detection and may not be reproducibly detected depending on the analytical sensitivity of the NAAT used. Furthermore, NAAT reagents may be in short supply when intensive care triaging is most important. Given the potential clinical sensitivity of SARS-CoV-2 antigenemia and its separate supply chain (13), we aimed to characterize the frequency, duration, and concentration of SARS-CoV-2 nucleocapsid antigen in plasma and its potential as a prognostic marker for COVID-19.

## Methods

### *Clinical Samples*

This retrospective study included a convenience set of individuals who received blood draws as part of routine clinical care from March to November 2020 at Stanford Healthcare, a tertiary-care academic hospital, along with its affiliated outpatient facilities in the San Francisco Bay Area. These individuals demonstrated either respiratory nucleic acid amplification testing (NAAT)-confirmed symptomatic COVID-19 (n=104), or SARS-CoV-2 NAAT-negative test result with either increased procalcitonin and/or C-reactive protein (n=47), or non-SARS-CoV-2 viral respiratory infections (rhinovirus n=3, human metapneumovirus n=1, adenovirus n=1). Remnant venipuncture blood samples were collected longitudinally when available from these individuals in K2EDTA, lithium heparin, or sodium heparin-coated vacutainers and stored at 4°C for up to 62 d. After centrifugation at 2,000 g for 8 min, plasma was separated and stored at -80°C in 2

mL screw cap micro tubes (Sarstedt, Newton, NC) for up to 350 d prior to assessment for antigen concentration. A subset of the specimens from the 104 NAAT-confirmed COVID-19 patients were used in prior studies (9,14).

Retrospective electronic medical record review was performed on all patients to collect demographic, laboratory, and encounter-related data. Non-critical inpatients were defined as having been hospitalized for >24 h at any point during the period of COVID-19-attributed illness. ICU admission was defined as having been provided intensive care or mechanical ventilation for any duration of time secondary to complications attributed to COVID-19 by the treating medical team on review of the electronic medical record. All deaths included in this study were attributed to COVID-19 by the treating medical team on review of the electronic medical record. The Research Electronic Data Capture (REDCap) platform was used to collect and manage the data. The Stanford University Institutional Review Board approved this study (Protocols IRB-48973 and IRB-55689), and individual patient consent was waived.

*Antigen Detection*

SARS-CoV-2 nucleocapsid antigen was quantified in plasma samples using an ultrasensitive antigen capture immunoassay platform, S-PLEX Direct Detection Assay, S-PLEX SARS-CoV-2 N Kit (Catalog #K150ADHS, Meso Scale Diagnostics [MSD], Rockville, MD), which was performed according to manufacturer instructions as previously described, and as detailed in the Supplemental Materials file (15).

Analytical validation was performed as detailed in the Supplemental Materials file (Supplemental Table 1, Supplemental Fig. 1). The test was interpreted as positive for antigen if the concentration exceeded a clinical cutoff of 2.80 $\log_{10}$ fg/mL. This threshold represented the 99$^{th}$ percentile, or mean+2.33*SD concentration of 80 pre-pandemic plasma samples from healthy blood donors. For samples with original concentrations above the upper limit of quantitation of 6.02 $\log_{10}$ fg/mL, the assay was repeated on a 1:100 dilution of the original sample in phosphate buffered saline. The individual performing the antigen testing was not blinded to the NAAT results or clinical information.

*Nucleic Acid Amplification Testing*

Respiratory NAAT results reported in this study were performed in the clinical virology laboratory as part of routine clinical care. A variety of methods were used including 1) a previously-described laboratory-developed reverse transcription quantitative polymerase chain reaction (RT-qPCR) targeting the envelope (*E*) gene on the Rotor-Gene Q (Qiagen, Germantown, MD) (16–18); 2) Xpert Xpress SARS-CoV-2 (Cepheid, Sunnyvale, CA), a rapid RT-qPCR method targeting both *E* and nucleocapsid (*N*) genes; 3) Panther Fusion SARS-CoV-2 (Hologic, Marlborough, MA), a high-throughput RT-qPCR method targeting open reading frame 1ab (*ORF1ab*); and 4) Aptima SARS-CoV2 (Panther System, Hologic), a transcription mediated amplification method targeting *ORF1ab*. All assays were conducted according to manufacturer and emergency authorization instructions (19–22). Cycle threshold ($C_t$) values were reported only for RT-qPCR methods. For Xpert Xpress, $C_t$ values were from the *E* gene target if available, and from the *N* gene target only if the *E* target was not detected.

*Antibody Testing*

A subset of the specimens had been previously tested for presence of anti-nucleocapsid (anti-N) IgG, IgM, and IgA using a laboratory-developed enzyme-linked immunosorbent assay (ELISA) as previously described (14). In brief, 96-well Corning Costar high binding plates (Thermo Fisher, Waltham, MA) were coated with recombinant SARS-CoV-2 nucleocapsid protein at a concentration of 0.1 μg per well overnight at 4°C and incubated with plasma at a 1:100 dilution for 1 hour at 37°C, with secondary detection by horseradish peroxidase conjugated goat anti-human IgG (γ-chain specific, catalog no. 62-8420, Thermo Fisher, 1:6,000 dilution), IgM (μ-chain specific, catalog no. A6907, Sigma, 1:6,000 dilution), or IgA (α-chain specific, catalog no. P0216, Agilent, 1:5,000 dilution). The positivity thresholds were set as previously described based on pre-pandemic samples: OD at 450 nm of ≥0.3 for IgG, ≥0.35 for IgM, and ≥0.1 for IgA (14).

*Statistical Analysis*

Only plasma samples taken ±1 day of first positive diagnostic respiratory NAAT from unique COVID-19 patients (n=74) along with specificity controls from SARS-CoV-2 respiratory NAAT-negative unique patients (n=52) were included in the evaluation of plasma antigen diagnostic performance. Clinical sensitivity and specificity were calculated using respiratory NAAT as the gold standard, and were reported with exact (Clopper-Pearson) 95% CI (23).

Wilcoxon rank sum and Kruskal-Wallis tests were used to compare median differences in antigen concentration and $C_t$ values among different groups of samples. All comparisons were two-sided with Type I error set at .05. No correction for multiple comparisons was performed

(24). Only the first sample from a unique individual within each week was included in each comparison, and for calculation of week by week sensitivity.

To further evaluate the relationship between plasma antigen concentration and disease severity, we performed uni- and multivariable logistic regression. *A priori* selected covariates included age, sex, diabetes, hypertension, obesity, and diagnostic respiratory sample $C_t$ value. The final multivariable model included only covariates with univariable $P<0.2$ in at least one comparison. Odds ratios (OR) were reported with 95% confidence intervals. Two-sided tests with $P<0.05$ were considered statistically significant. Analysis was performed using JMP Statistical Software Version 14 (SAS Institute, Cary, NC).

The linear mixed effects models were constructed using R packages lme4, ggeffects, MuMIn, and stargazer, and included only data points between 5-40 d after symptom onset ($d$) due to non-linearity, non-normality, and heteroskedasticity of $\log_{10}$ antigen concentration in fg/mL ($a$) beyond this time frame (25). Outpatient samples were excluded from the model due to paucity of longitudinal samples in these individuals. Model A included only random intercept of patient identity ($p$), with formula: $\log_{10}(a) \sim (1|p)$. Model B included random intercept and slope of patient identity with fixed effect of $\log_{10}(d)$: $\log_{10}(a) \sim \log_{10}(d) + (1+ \log_{10}(d)|p)$. Model C included random intercept and slope of patient identity with fixed effects of both $\log_{10}(d)$ and severity ($s$): $\log_{10}(a) \sim \log_{10}(d) + s + (1+ \log_{10}(d)|p)$, and was used to create Fig. 3 and Supplemental Fig. 3. All statistical analysis and figures were performed/created using R 4.0.2.

**Results**

The 104 individuals with COVID-19 included in this study were 50% female (n=52), had a median age of 57 y (IQR 39-72), and were comprised of 22% outpatients (n=23), 41% non-critical inpatients (n=42), and 38% ICU patients (n=39). Among these individuals, 74 had available plasma samples taken ±1 d of their first positive (diagnostic) respiratory NAAT, which was a median of 7 d (IQR 3-10) from symptom onset (Table 1). Plasma nucleocapsid antigen was detected using a commercial kit in 68/74 individuals at this time point, resulting in a clinical sensitivity of 91.9% (95% CI 83.2-97.0%) for COVID-19 diagnosis. Sensitivity was similar in outpatients, non-critical inpatients, and ICU patients (Supplemental Table 2).

Among the 68 antigen-positive individuals, plasma antigen concentration at diagnosis appeared lowest among outpatients (median [IQR] of 5.4 $\log_{10}$ fg/mL [3.9-6.0]), followed by non-ICU inpatients (6.0 [5.4-6.5]), and highest in ICU patients (6.6 [6.1-7.2], Kruskal-Wallis $P$<.001) (Fig. 1A). In contrast, respiratory RT-qPCR $C_t$ values from a variety of methods/targets were not significantly different between these groups (Fig. 1B). These methods/targets included GeneXpert Xpress E gene (21/74, 28%), laboratory-developed test targeting *E* gene (30/74, 41%), and Panther Fusion targeting *ORF1ab* (23/74, 31%). Analyzing the data by respiratory NAAT platform and target did not reveal a stronger linear correlation between respiratory $C_t$ and log-transformed plasma antigen values (Supplemental Figure 2).

Individuals with antigen-negative plasma samples had a later median respiratory RT-qPCR $C_t$ than did their antigen-positive counterparts (25.8 [IQR 18.6-30.9] vs. 38.2 [IQR 37.4-39.2], $P$<.001) (Fig. 1C). One sample was from an outpatient who was already anti-N IgG positive, IgA

positive, and IgM negative at the time of diagnostic respiratory NAAT, which was taken 2 days after onset of a mild sore throat with no other symptoms. The remaining 5 individuals never seroconverted for any Ig isotype. Three of these antigen-negative individuals who never seroconverted were diagnosed via Panther Fusion targeting *ORF1ab*, and two were diagnosed via the laboratory-developed test targeting the *E* gene.

In univariable logistic regression, plasma antigen concentration at diagnosis was associated with ICU relative to non-ICU inpatient status (*P*=.02), non-ICU inpatient relative to outpatient status (*P*=.04), and ICU relative to outpatient status (*P*<.001). After adjusting for age, sex, and comorbidities, a one unit increase in $\log_{10}$ antigen concentration was associated with an OR of 2.8 (95% CI 1.2-6.2, *P*=.01) for ICU admission relative to non-ICU inpatient admission, and an OR of 4.2 (95% CI 1.7-10.3, *P*=.002) for ICU admission relative to outpatient status (Supplemental Table 3). For example, the adjusted risk of ICU admission increased from 16% to 39% with an increase from 4 $\log_{10}$ fg/mL to 5 $\log_{10}$ fg/mL plasma antigen, with a concomitant decrease in non-ICU inpatient status and outpatient disposition of 40% to 36% and 44% to 26%, respectively. Plasma antigen concentration, however, was not independently associated with non-ICU inpatient status vs. outpatient status after multivariable regression.

The 52 individuals negative for SARS-CoV-2 by respiratory NAAT, included as specificity controls, were 42% female (n=22), had a median age of 54 y (20-67), and were comprised of 2% outpatients (n=1), 40% inpatients (n=21), 58% ICU patients (n=30). Plasma antigen was above the threshold of positivity in 3/52 individuals, resulting in a clinical specificity of 94.2% (95% CI 84.1-98.8%). All 3 false-positive specimens had antigen concentrations (2.82, 3.00, 3.01 $\log_{10}$

fg/mL) near the positivity threshold of 2.80 $log_{10}$ fg/mL. One individual was hospitalized for cellulitis and was included as a negative control due to increased C-reactive protein. Another was an ICU patient with myocardial infarction with increased C-reactive protein and procalcitonin of unclear etiology. The third was an infant with rhinovirus bronchiolitis.

To evaluate whether plasma antigen concentration at later time points was associated with disease severity or death, 777 available longitudinally-collected plasma samples from all 104 individuals with COVID-19 were tested. There was a median of 5 (IQR 2-10) specimens per individual available, collected a median of 18 d (IQR 11-52) days after symptom onset. Assay sensitivity for COVID-19 diagnosis was maintained through the first two weeks after symptom onset, then steadily decreased thereafter (Table 2). While outpatient plasma antigen concentrations were lower than those of hospitalized individuals within the first week after symptom onset (median [IQR] of 5.3 [4.9-5.8] vs. 6.3 [5.4-7.0], $P$=.006), this was not true for subsequent weeks (Fig. 2). Within the first week, only outpatient vs. inpatient (median [IQR] of 5.3 [4.9-5.8] vs. 6.3 [5.4-6.7] $log_{10}$ fg/mL, $P$=.02) and outpatient vs. ICU (median [IQR] of 5.3 [4.9-5.8] vs. 6.5 [5.6-7.1] $log_{10}$ fg/mL, $P$=.008) each-pair comparisons were statistically significant. There were 25 deaths due to COVID-19. Comparing results from these individuals to those who survived, there was no detectable difference in plasma antigen (Supplemental Fig. 3). There was also no significant difference in plasma antigen concentrations by week, either in K2EDTA vs. heparinized plasma, or by number of days stored at 4°C prior to centrifugation (Supplemental Fig. 4).

To determine whether rate of plasma antigen decrease over time was associated with disease severity, a linear mixed-effects model was constructed (Supplemental Fig. 5). From days 5-40 after symptom onset, plasma antigen concentration decreased linearly in relation to $\log_{10}$-transformed days after symptom onset (β = -7.9 [95% CI -9.2 to -6.5]) (Fig. 3). Non-critical inpatient vs. ICU status was not independently associated with change in antigen concentration over time (β = 0.2 [95% CI -0.5 to 0.9]) (Supplemental Table 4).

Individual plots of longitudinal plasma antigen concentration, respiratory RT-qPCR $C_t$, and anti-N indices, particularly IgG, demonstrated general concordance between time of antigen disappearance and peak antibody titers (Fig. 4A, B, Supplemental Figure 6). Respiratory RT-qPCR $C_t$ values also appeared to generally correlate with plasma antigen concentrations, except in several cases of positive RT-qPCRs with non-detectable plasma antigen following apparent respiratory RNA clearance >1 month prior (Figure 4C-E). All of these individuals were immunocompromised secondary to active malignancy, chemotherapy, and/or solid-organ transplant, and had no documented new SARS-CoV-2 exposure.

**Discussion**

There are limited data describing the diagnostic and prognostic value of SARS-CoV-2 nucleocapsid antigenemia. In this study, we observed >90% diagnostic sensitivity and specificity, and an association between higher plasma antigen concentration and increased disease severity at time of diagnosis and within the first week of symptom onset.

Two prior studies have examined a single molecular array bead-based ELISA (SIMOA) for SARS-CoV-2 nucleocapsid antigenemia, and have reported clinical sensitivities lower than that observed in our study (64-74%), despite a reported limit of detection 10-fold lower than MSD S-PLEX (26,27). As such, this difference may be due to inclusion of a greater proportion of patients with mild disease and/or plasma samples further out from diagnosis. In contrast, one study reported a clinical sensitivity of 93% within 2 weeks of symptom onset for the COVID Quantigene ELISA, which is more consistent with our observations (13). Interestingly, nucleocapsid antigenemia had similar sensitivity for diagnosis of SARS-CoV-1 during the original 2003 SARS outbreak (28,29). Similar to this other study (13), we also found that individuals negative for plasma antigen at diagnosis had later median respiratory RT-qPCR $C_t$ values, or lower viral burden, and that clinical sensitivity declined beyond 14 d after symptom onset.

Because a separate study described a greater proportion of false-positives in sick pre-pandemic individuals than in their healthy counterparts (27), we purposefully included predominantly hospitalized individuals with increased inflammatory markers in our assessment of assay specificity. As such, the clinical specificity of 94.2% (95% CI 84.1-98.8%) reported in our limited cohort of 52 controls may represent an over- or underestimation of specificity in other patient populations. Laboratories utilizing this assay should consider using a local reference population-based threshold rather than the assay's limit of detection, or the threshold reported in this study. Of note, all false-positives in our study were near the threshold of positivity, suggesting that orthogonal testing of borderline specimens (e.g., between 2.6-3.2 $\log_{10}$ fg/mL) or collection of a second specimen for additional testing may help increase clinical specificity.

Further studies with larger numbers of more heterogeneous controls will be necessary to establish whether this assay could be specific enough to be used for primary diagnosis.

Similar to the two studies discussed above (13, 27*),* we observed that plasma antigen may also have value in prognostication, though these studies did not attempt to adjust for possible confounding demographic factors and comorbidities, or determine whether rate of antigenemia decline was associated with disease severity. In our study, among diagnostic specimens (±1 d of first positive NAAT), collected a median of 7 d (IQR 3-10) after symptom onset, plasma antigen concentration was higher in patients requiring ICU admission, even after adjusting for age, sex, and comorbidities. As such, a quantitative nucleocapsid antigenemia test at the time of diagnosis, especially within the first week of symptom onset, may have potential value in triaging patients for higher-level care, though its added value relative to other biomarkers such as RNAemia will need to be evaluated (9,30).

Based on our mixed-effects model, we found no relationship between rate of antigen decrease and disease severity. This suggests that nucleocapsid plasma antigen may serve as a biomarker of tissue damage and vascular leakage, rather than as a driver of infection or disease response (31). As such, longitudinal monitoring of plasma antigen concentrations is unlikely to be helpful in predicting outcomes or response to therapy.

Nucleocapsid antigenemia, however, does appear to correlate with anti-nucleocapsid antibody seroconversion. The majority of individuals negative for nucleocapsid antigen at time of diagnosis never seroconverted, in contrast to antigen-positive individuals, almost all of whom eventually developed at least one anti-nucleocapsid antibody isotype. This raises the possibility that at least some of the individuals with false-negative plasma antigen may have actually had a false-positive NAAT.

Strengths of this study include the testing of a large set of 777 specimens across 104 patients with COVID-19 and adequate follow-up using a novel method that offers similar sensitivity as nasopharyngeal NAAT in patients with respiratory viral burdens at highest risk of onward transmission (32–34). As a high-throughput approach ideal for implementation in a high-complexity laboratory, this assay would not fill the same niche as less sensitive point-of-care rapid antigen tests (35). Rather, this assay could be employed as a qualitative diagnostic during times of respiratory specimen collection or NAAT supply chain shortages, or to provide both diagnostic and antibody testing on a single sample. At the time of initial diagnosis within the first week of symptom onset, quantitative nucleocapsid antigen testing may also have utility in prognostication (outpatient vs. non-critical inpatient vs. ICU) that outperforms nasopharyngeal RT-qPCR Ct values. As such, it could be useful as an adjunct to clinical decision algorithms employing patient-specific demographic and laboratory measures.

Limitations of this study include the testing of remnant blood samples in a non-consecutive symptomatic population, which may have introduced selection bias. Furthermore, only a limited number of individuals were included from each admission category, which limits power to detect a difference between the groups if present. The use of remnant blood samples may also have introduced variability based on storage and collection conditions, though we found no difference in antigen concentrations by week in K2EDTA vs. heparinized samples, or by storage time.

Future prospective trials with larger numbers and standardized sample collection protocols will be necessary to validate the diagnostic and prognostic value of this platform, especially in outpatients and asymptomatic individuals. These trials should ideally evaluate other severity variables and complications we were not powered to assess including oxygen needs, mechanical ventilation, therapy, and time since symptom onset. Future studies may also compare RNAemia with quantative antigenemia to determine whether these two potential biomarker assays might offer adjunctive versus identical prognostic information.

As this study was conducted prior to wide availability of vaccination and circulation of variants of interest/concern, it is possible that the diagnostic and prognostic value of nucleocapsid antigenemia may be different in the current population, though this is less likely as vaccination and variant mutations have largely involved the spike protein. Lastly, because we did not have samples from individuals with non-SARS-CoV-2 seasonal coronaviruses, we cannot exclude the possibility of cross-reactivity in this setting.

In summary, plasma SARS-CoV-2 antigen detection had >90% clinical sensitivity and specificity for COVID-19 diagnosis within 2 weeks of symptom onset in this retrospective cohort study. It also demonstrated potential as a prognostic assay to identify patients more likely to require intensive care.

**Acknowledgments**

We thank the Stanford Clinical Virology, Chemistry, and Hematology Laboratory staff for their hard work and dedication to patient care, and for their assistance in saving the remanant blood samples that made this work possible.

**Author Contributions:** *All authors confirmed they have contributed to the intellectual content of this paper and have met the following 4 requirements: (a) significant contributions to the conception and design, acquisition of data, or analysis and interpretation of data; (b) drafting or revising the article for intellectual content; (c) final approval of the published article; and (d) agreement to be accountable for all aspects of the article thus ensuring that questions related to the accuracy or integrity of any part of the article are appropriately investigated and resolved.*

**Authors' Disclosures or Potential Conflicts of Interest:** *Upon manuscript submission, all authors completed the author disclosure form. Disclosures and/or potential conflicts of interest:*

**Employment or Leadership:** None declared.
**Consultant or Advisory Role:** None declared.
**Stock Ownership:** None declared.
**Honoraria:** None declared.
**Research Funding:** This work was supported by Meso Scale Diagnostics (MSD), who provided the S-PLEX SARS-CoV-2 N Kits, BioTek 405 Select automated 96-well plate washer, and MESO® SECTOR S 600 Reader used in this study. The Stanford REDCap platform (http://redcap.stanford.edu) is developed and operated by Stanford Medicine Research IT team. The REDCap platform services at Stanford are subsidized by a) Stanford School of Medicine Research Office, and b) the National Center for Research Resources and the National Center for Advancing Translational Sciences, National Institutes of Health, through grant UL1 TR001085. Portions of this work were supported by NIH/NIAID R01AI127877 (S.D. Boyd), NIH/NIAID R01AI130398 (S.D. Boyd), NIH 1U54CA260517 (S.D. Boyd and B.A. Pinsky), an endowment from the Crown Family Foundation (S.D. Boyd), and a Coulter COVID-19 Rapid Response Award (S.D. Boyd). C.A. Hogan, grant from Genome BC/Michael Smith Foundation for Health Research/BCCDC Foundation to institution.
**Expert Testimony:** None declared.
**Patents:** None declared.

**Role of Sponsor:** The funding organizations played no role in the design of study, choice of enrolled patients, review and interpretation of data, preparation of manuscript, or final approval of manuscript.

**Tables**

**Table 1.** Demographics, comorbidities, and plasma SARS-CoV-2 nucleocapsid antigen concentrations for individuals with plasma samples drawn ±1 day from diagnostic respiratory NAAT (n=74).

| Covariate[a] | Overall (n=74) | Outpatient (n=18) | Inpatient[b] (n=30) | ICU (n=26) | *P* value[c] |
|---|---|---|---|---|---|
| Age, y | 56 (37-75) | 43 (31-63) | 60 (39-82) | 61 (40-72) | .07 |
| Sex (Female) | 39 (52.7%) | 10 (55.6%) | 19 (63.3%) | 10 (38.5%) | .2 |
| Diabetes | 27 (36.5%) | 4 (22.2%) | 10 (33.3%) | 13 (50.0%) | .1 |
| Hypertension | 31 (41.9%) | 5 (27.8%) | 11 (36.7%) | 15 (57.7%) | .1 |
| Obesity | 31 (41.9%) | 6 (33.3%) | 13 (43.3%) | 12 (46.2%) | .7 |
| Days Sx | 7 (3-10) | 7 (4-10) | 5 (2-10) | 8 (3-11) | .3 |
| Resp. $C_t$ | 25.8 (18.6-30.9) | 28.4 (16.3-33.2) | 25.8 (18.7-31.1) | 23.4 (19.1-29.4) | .6 |
| $Log_{10}$ Ag | 6.2 (5.4-6.7) | 5.4 (3.9-6.0) | 6.0 (5.4-6.5) | 6.6 (6.1-7.2) | < .001 |

SARS-CoV-2, severe acute respiratory syndrome coronavirus 2; NAAT, nucleic acid amplification test; ICU, intensive care unit; Days Sx, days from symptom onset; Resp. $C_t$, respiratory NAAT cycle threshold; $Log_{10}$ Ag, $Log_{10}$ nucleocapsid antigen concentration (fg/mL)

[a] Continuous variables reported as median (interquartile range); categorical variables reported as N (%)

[b] Inpatients excluding those requiring intensive care

[c] Kruskal-Wallis test

**Table 2**. Clinical sensitivity of SARS-CoV-2 nucleocapsid antigen for the diagnosis of COVID-19 in plasma samples, subset by weeks from symptom onset.

| Week After Symptom Onset[a] | Plasma SARS-CoV-2 Nucleocapsid Antigen | | | |
|---|---|---|---|---|
| | Positive | Negative | Total | Sensitivity (95% CI) |
| 1 | 44 | 5 | 49 | 89.8% (77.8-96.6%) |
| 2 | 64 | 6 | 70 | 91.4% (82.3-96.8%) |
| 3 | 35 | 12 | 47 | 74.5% (59.7-86.1%) |
| 4 | 16 | 14 | 30 | 53.3% (34.3-71.7%) |
| 5 | 9 | 15 | 24 | 37.5% (18.8-59.4%) |
| 6 | 8 | 14 | 22 | 36.4% (17.2-59.3%) |
| 7+ | 5 | 30 | 35 | 14.3% (4.8-30.26%) |

SARS-CoV-2, severe acute respiratory syndrome coronavirus 2; COVID-19, coronavirus disease 2019; CI, confidence interval

[a]If an individual had more than one plasma sample taken within the same week, only the first one was included. As such, each row contains only one sample per individual.

## Figure Legends

**Figure 1.** Comparison of SARS-CoV-2 nucleocapsid antigen concentration in plasma, diagnostic respiratory swab cycle threshold ($C_t$), and disease severity in 74 plasma samples drawn within ±1 d of diagnostic respiratory reverse transcription quantitative polymerase chain reaction. The dashed line represents the positivity threshold. Statistical significance from two-sided Wilcoxon rank sum testing is denoted as ns: $P$ >.05, *: $P$ ≤.05, **: $P$ ≤.01, ***: $P$ ≤.001, ****: $P$≤.0001.

**Figure 2.** A-D, Comparison of plasma SARS-CoV-2 nucleocapsid antigen concentration by days from symptom onset, subgrouped by disease severity, in 777 plasma samples from 104 unique individuals with respiratory NAAT-confirmed COVID-19. These samples/individuals include the 74 samples/individuals from Fig. 1. E, Boxplots of the first positive sample from each unique individual each week after symptom onset. The dashed line represents the positivity threshold. Statistical significance from Kruskal-Wallis testing is denoted as ns: $P$ >.05, *: $P$ ≤.05, **: $P$ ≤.01, ***: $P$ ≤.001.

**Figure 3.** Linear mixed-effects model of plasma SARS-CoV-2 nucleocapsid antigen concentration based on days from symptom onset and disease severity, accounting for inter-individual random variation, in 388 inpatient and ICU plasma samples collected between 5-40 days after symptom onset. The points represent observed values, while the lines represent predicted values. Inpatient vs. ICU disease severity was not significantly associated with time-dependent antigen concentration, as evidenced by the overlap between shaded 95% confidence intervals (A). Inter-individual random variation (both slope and intercept) accounted for the majority of the remaining variance, with each color representing a different individual (B). The dashed line represents the positivity threshold.

**Figure 4.** Individual timelines of plasma SARS-CoV-2 nucleocapsid antigen concentration (top panel, green diamond, left side Y-axis), respiratory swab reverse transcription quantitative polymerase chain reaction (RT-qPCR) cycle threshold ($C_t$) values (top, orange + for positive RT-qPCR, orange x for negative RT-qPCR, right side inverted Y-axis), and anti-nucleocapsid antibody concentrations (bottom panel, red circle for IgA, blue square for IgG, purple triangle for IgM), by days from symptom onset. The dashed horizontal line represents threshold of positivity for both plasma antigen and respiratory RT-qPCR. Inpatient #2 and Intensive Care Unit Patient (ICU) #9 are examples of the majority of antigen-positive individuals whose seroconversion coincided with disappearance of plasma antigen (A-B). Inpatient #4 and #5, and ICU #2 all had positive respiratory RT-qPCRs >1 month after earlier viral RNA clearance, without reappearance of plasma antigen (C-E). Plots for all 83 individuals with >1 plasma sample in this study can be found in Supplemental Fig. 6.

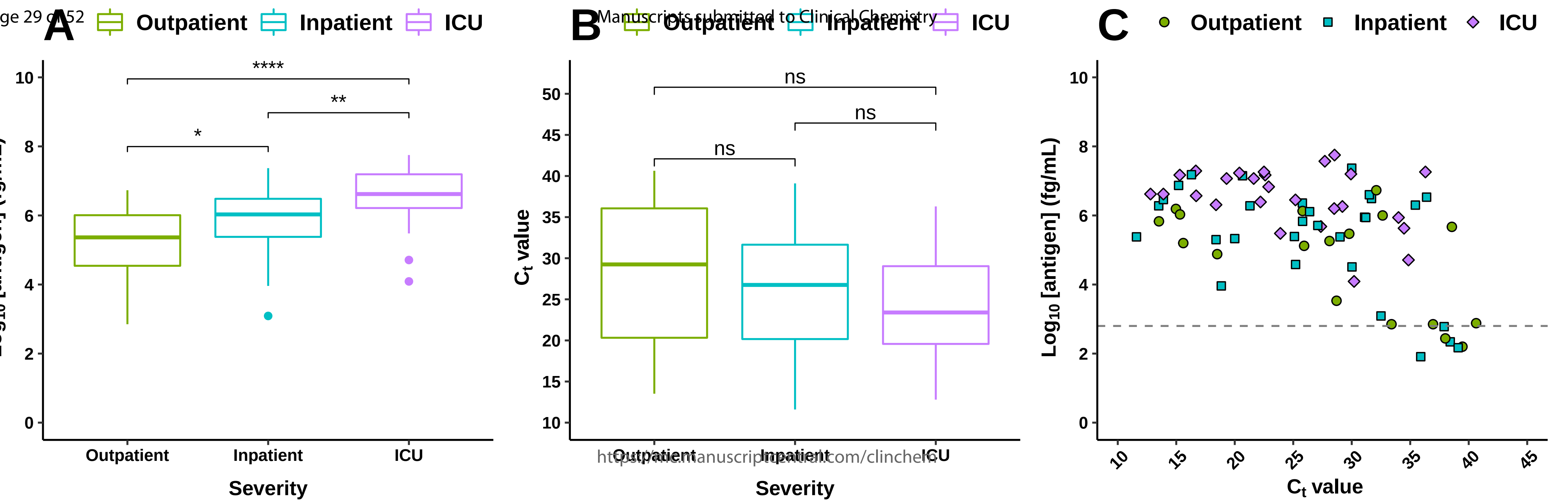

A
Outpatient
Inpatient
ICU
Log10 [antigen] (fg/mL)
****
**
*
Outpatient
Inpatient
ICU
Severity
B
Outpatient
Inpatient
ICU
Ct value
ns
ns
ns
Outpatient
Inpatient
ICU
Severity
C
Outpatient
Inpatient
ICU
Log10 [antigen] (fg/mL)
Ct value

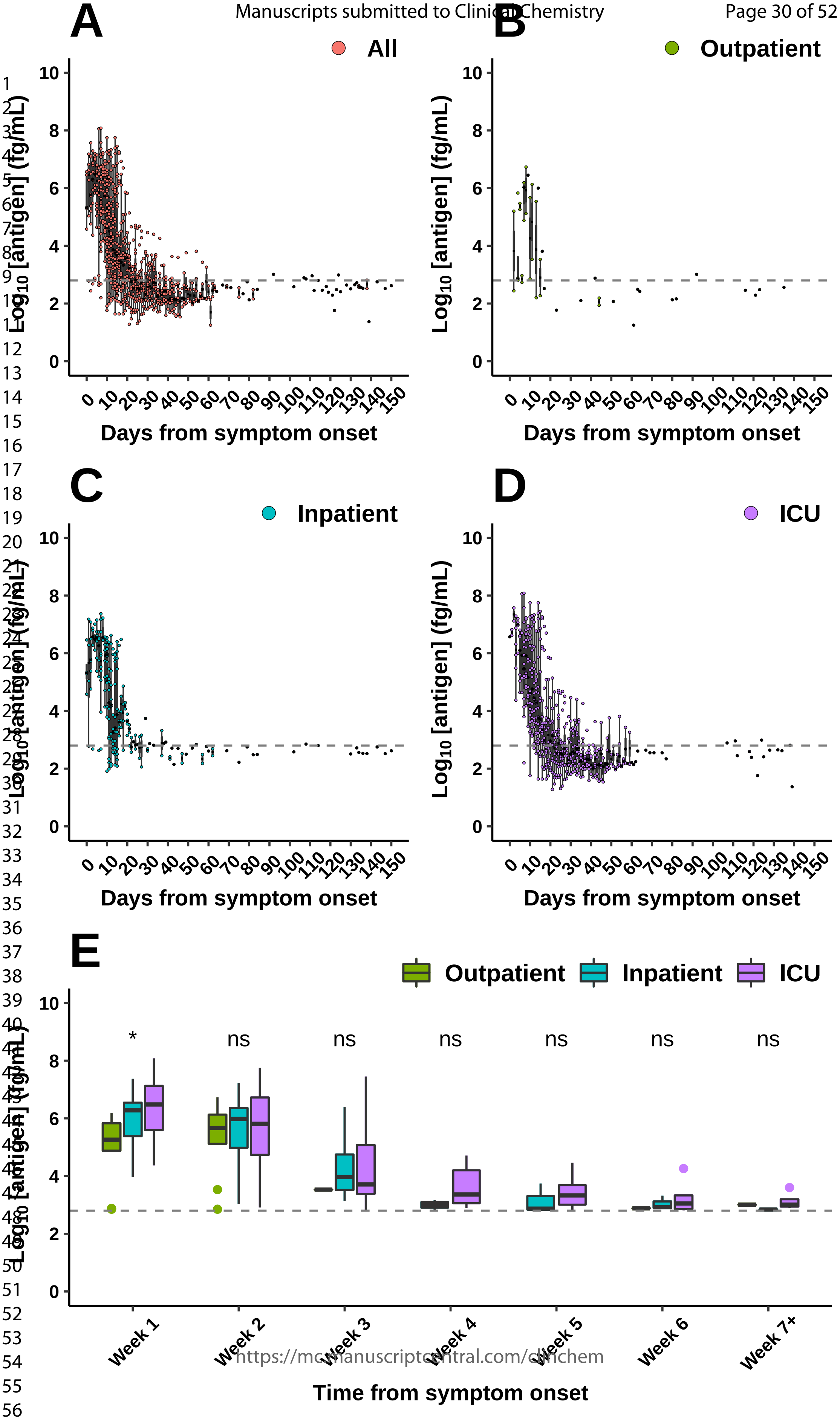

A
All
B
Outpatient
C
Inpatient
D
ICU
Log10 [antigen] (fg/mL)
Days from symptom onset
E
Outpatient
Inpatient
ICU
*
ns
ns
ns
ns
ns
ns
Week 1
Week 2
Week 3
Week 4
Week 5
Week 6
Week 7+

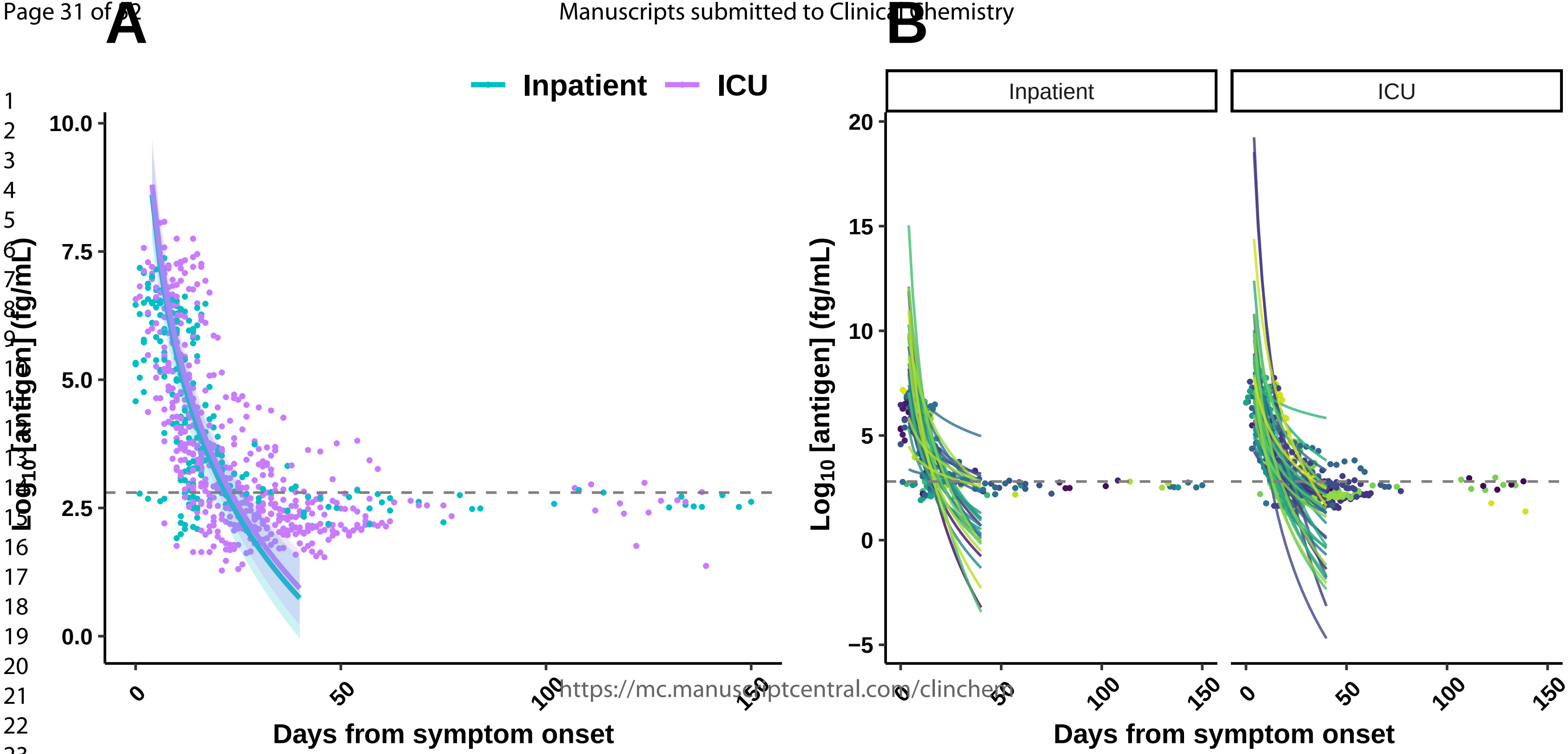

Time from symptom onset https://mc.manuscriptcentral.com/clinchem

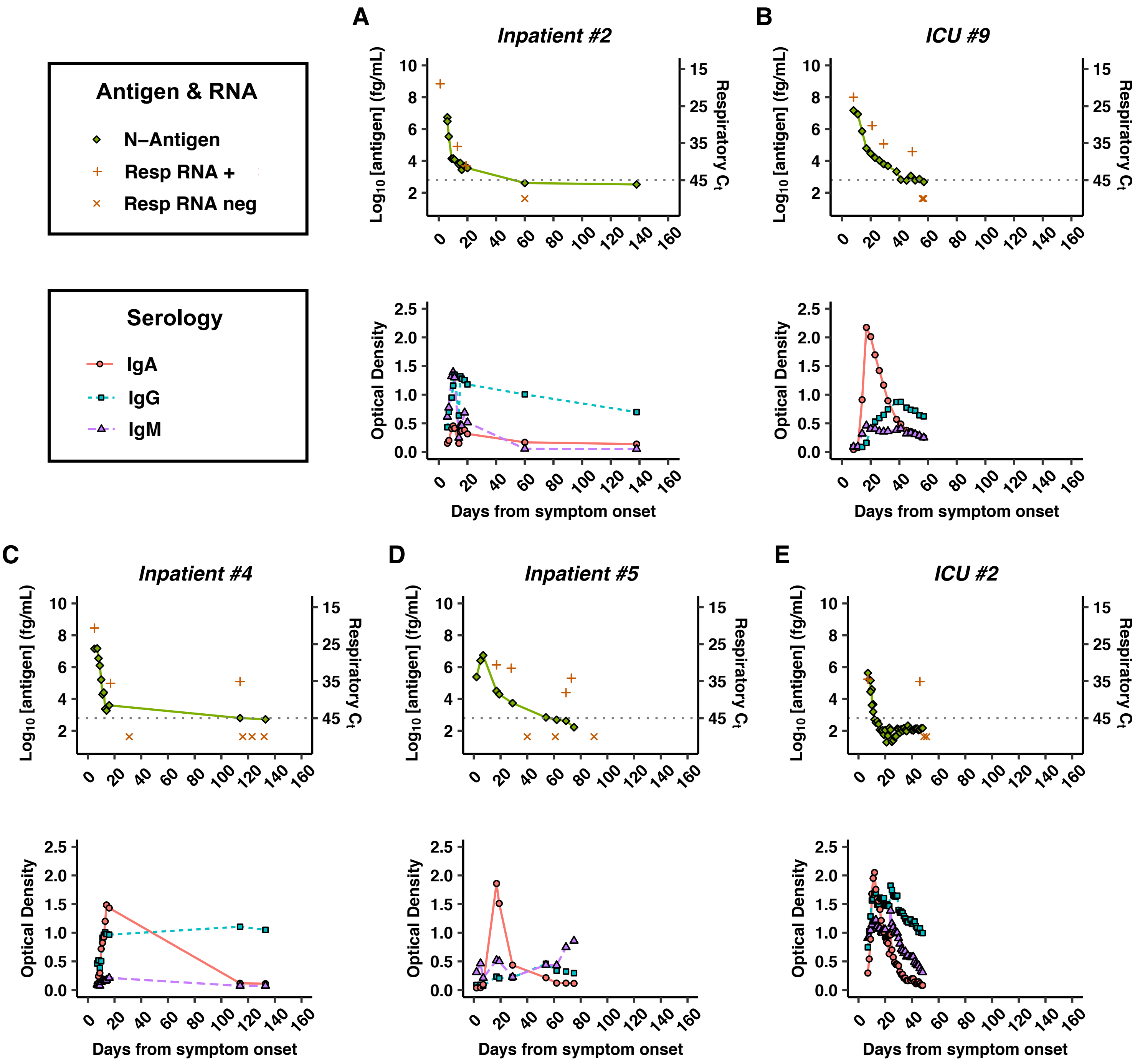

Antigen & RNA
N–Antigen
Resp RNA +
Resp RNA neg
Serology
IgA
IgG
IgM
A
Inpatient #2
B
ICU #9
C
Inpatient #4
D
Inpatient #5
E
ICU #2
Log10 [antigen] (fg/mL)
Respiratory Ct
Optical Density
Days from symptom onset

## Supplementary Material

hvab216_Supplementary_Data

