# Supplementary material for: SARS-CoV-2 Nucleocapsid Plasma Antigen for Diagnosis and Monitoring of COVID-19
Source: Clin Chem. 2021 Oct 4;68(1):204–13. doi: 10.1093/clinchem/hvab216 (PMC8522398; doi:10.1093/clinchem/hvab216)
Supplement: hvab216_Supplementary_Data [file hvab216_supplementary_data.zip › MSDP_ClinChemRev3_Supp_082921_reviewed_bap09052021.docx]

**Supplemental Materials**

*Antigen Detection*

First, S-PLEX 96-well SECTOR plates were coated with biotinylated capture antibodies and incubated either for 1 hour at room temperature (RT) or overnight at 4ºC. Immediately after plates were blocked using the MSD blocking solution, 25 µL of sample was added to each well. A 7-point calibration curve and negative control consisting of assay diluent was run in duplicate on each plate. Following sample incubation at RT for 1.5 hours, detection antibody was added (MSD TURBO-BOOST™ Detection Antibody) and incubated for 1 h at RT, and an enhancement step was performed by using the MSD S-PLEX Enhance solution with a 30-min incubation at RT. Subsequently, detection solution (TURBO-TAG™ Detection Solution) was added. After incubation for 1 h at 27ºC and addition of MSD GOLD™ Read Buffer B, the plates were read using a MESO® SECTOR S 600 Reader.

All incubations were performed in a plate shaker at 700 rotations per minute. Each incubation step was followed by a washing step, consisting of three washes in MSD Tris Wash Buffer using the BioTek 405 Select automated 96-well plate washer (BioTek, Winooski, VT). Raw signal was converted to a concentration based on linear regression to the 7-point calibration curve, which was run in duplicate on each plate. The total turnaround time of testing was five hours, or four hours if the coating step was performed overnight at 4ºC in advance.

*Analytical Validation*

The manufacturer-provided 8-point calibration set was run in duplicate on S-PLEX across 20 different runs to determine intra-assay, inter-assay, intra-laboratory precision, and total coefficient of variation. The pre-specified acceptable total coefficient of variation was set at 20%. The assay limit of blank and limit of detection were calculated from the mean and standard deviation of the log_10_ concentration of manufacturer-provided blank negative controls (Standard 8) and lowest concentration positive controls (Standard 7) over 40 repeats across 20 different runs per CLSI EP17 recommendations. Manufacturer-provided nominal concentrations of the calibrator material were available for 17/20 of these runs, and were used to conduct Passing-Bablok regression and calculate mean bias. The log_10_ value was used rather than the raw concentration, as these values better approximated a normal distribution.

Analytical validation established limit of blank at 2.06 log_10_ fg/mL, limit of detection at 2.31 log_10_ fg/mL, lower limit of quantification at 2.44 log_10_ fg/mL, and upper limit of quantification at 6.02 log_10_ fg/mL in our laboratory (Supplemental Table 1). Passing-Bablok regression demonstrated no significant fixed or proportional bias, with slope and intercepts having 95% confidence intervals (CI) crossing 1 and 0 respectively (Supplemental Fig. 1A). Mean bias was 0.01 log_10_ fg/mL (95% CI -0.1 to 0.11 log_10_ fg/mL (Supplemental Fig. 1B).

**Supplemental Tables**

**Supplemental Table 1.** Intra-assay, inter-assay, intra-laboratory precision, and total coefficient of variation, for S-PLEX SARS-CoV-2 nucleocapsid antigen calibrators, run in duplicate across 20 runs.

| **Standard** | **Mean log_10_ antigen concentration (fg/mL)** | **S_r_** | **S_b_** | **S_l_** | **Total CV (%)** |
| --- | --- | --- | --- | --- | --- |
| 1 | 6.02 | 0.02 | 0.03 | 0.04 | 0.6 |
| 2 | 5.45 | 0.03 | 0.03 | 0.04 | 0.8 |
| 3 | 4.81 | 0.04 | 0.04 | 0.05 | 1.0 |
| 4 | 4.18 | 0.05 | 0.04 | 0.05 | 1.2 |
| 5 | 3.64 | 0.05 | 0.05 | 0.06 | 1.7 |
| 6 | 3.01 | 0.03 | 0.06 | 0.07 | 2.2 |
| 7 | 2.44 | 0.06 | 0.07 | 0.08 | 3.2 |
| SARS-CoV-2, severe acute respiratory syndrome coronavirus 2; S_r_, intra-assay precision; S_b_, inter-assay precision; S_l_, intra-laboratory precision; CV, coefficient of variation | | | | | |

**Supplemental Table 2.** Clinical sensitivity of SARS-CoV-2 nucleocapsid antigen for the diagnosis of COVID-19 in plasma samples from unique individuals drawn ±1 d from diagnostic respiratory NAAT (n=74).

| Category | Subcategory | Plasma SARS-CoV-2 Nucleocapsid Antigen | | | |
| --- | --- | --- | --- | --- | --- |
|  |  | Positive | Negative | Total | Sensitivity (95% CI) |
| Total | Total | 68 | 6 | 74 | 91.9% (83.2-97.0%) |
| By Severity | Outpatient | 16 | 2 | 18 | 88.9% (65.3-98.6%) |
|  | Inpatient (non-ICU) | 26 | 4 | 30 | 86.7% (69.3-96.2%) |
|  | ICU | 26 | 0 | 26 | 100.0% (86.8-100.0%) |
| SARS-CoV-2, severe acute respiratory syndrome coronavirus 2; COVID-19, coronavirus disease 2019; CI, confidence interval; ICU, intensive care unit | | | | | |

**Supplemental Table 3.** Univariable and multivariable logistic regression for disease severity in individuals positive for plasma SARS-CoV-2 nucleocapsid antigen within ±1 d from diagnostic respiratory NAAT (n=68).

| Comparison | Covariate | Univariable | Multivariable | |
| --- | --- | --- | --- | --- |
|  |  | *P* Value | Odds Ratio | *P* Value |
| ICU vs. Inpatient | Log_10_ Ag | .02 | 2.8 (1.2-6.2) | .01 |
|  | Age | .5 | 1.0 (0.9-1.0) | .08 |
|  | Sex (Female) | .06 | 0.5 (0.2-0.9) | .03 |
|  | Diabetes | .4 | 1.3 (0.6-2.7) | .5 |
|  | Hypertension | .1 | 1.2 (0.6-2.7) | .6 |
|  | Obesity | .8 | - | - |
|  | Resp. Ct | .9 | - | - |
| Inpatient vs. Outpatient | Log_10_ Ag | .04 | 1.5 (0.8-3.0) | .2 |
|  | Age | .03 | 1.0 (1.0-1.1) | .1 |
|  | Sex (Female) | .3 | 1.3 (0.6-2.6) | .5 |
|  | Diabetes | .2 | 1.7 (0.7-3.9) | .3 |
|  | Hypertension | .5 | 0.7 (0.3-1.6) | .4 |
|  | Obesity | .8 | - | - |
|  | Resp. Ct | .4 | - | - |
| ICU vs. Outpatient | Log_10_ Ag | < .001 | 4.2 (1.7-10.3) | .002 |
|  | Age | .1 | 1.0 (1.0-1.0) | .9 |
|  | Sex (Female) | .5 | 0.6 (0.3-1.3) | .2 |
|  | Diabetes | .05 | 2.1 (0.8-5.6) | .1 |
|  | Hypertension | .04 | 0.8 (0.3-2.2) | .7 |
|  | Obesity | .6 | - | - |
|  | Resp. Ct | .3 | - | - |
| SARS-CoV-2, severe acute respiratory syndrome coronavirus 2; NAAT, nucleic acid amplification test; ICU, intensive care unit; Log_10_ Ag, Log_10_ nucleocapsid antigen concentration (fg/mL); Resp. C_t_, respiratory NAAT cycle threshold | | | | |

**Supplemental Table 4.** Linear mixed-effects model for SARS-CoV-2 nucleocapsid antigen concentration in samples obtained between 5-40 d from symptom onset, accounting for inter-individual random variation (n=388).

| Model Parameters | Linear Mixed-Effects Model Estimates (95% CI) | | |
| --- | --- | --- | --- |
|  | (A) | (B) | (C) |
| Log_10_ (Days Sx Onset) |  | -7.9^***^ (-9.2, -6.5) | -7.9^***^ (-9.2, -6.5) |
| Severity: ICU |  |  | 0.2 (-0.5, 0.9) |
| Constant | 4.7^***^ (4.5, 4.9) | 13.4^***^ (11.8, 15.1) | 13.35^***^ (11.7, 15.0) |
| Observations | 388 | 388 | 388 |
| Log Likelihood | -648.1 | -390.3 | -390.3 |
| Akaike Inf. Crit. | 1302.2 | 792.6 | 794.5 |
| Bayesian Inf. Crit. | 1314.0 | 816.4 | 822.3 |
| Marginal R^2^ | 0 | 0.47 | 0.46 |
| Conditional R^2^ | 0.35 | 0.97 | 0.97 |
| SARS-CoV-2, severe acute respiratory syndrome coronavirus 2; CI, confidence interval; Sx, symptom; ICU, intensive care unit; Inf. Crit., Information Criterion; R^2^, coefficient of determination for generalized linear mixed model; ^***^p<0.001  Model (A) includes only random intercept of patient identity  Model (B) includes random intercept and slope of patient identity with fixed effect of log_10_ days from symptom onset  Model (C) includes random intercept and slope of patient identity with fixed effects of both log_10_ days from symptom onset and severity (non-critical inpatient vs. ICU) | | | |

**Supplemental Figure 1.** Passing-Bablok regression (A) and Bland-Altman plot (B) for nominal versus measured log_10_ antigen concentration (fg/mL) on manufacturer-provided recombinant SARS-CoV-2 nucleocapsid antigen calibrators at 7 different known concentrations spanning the assay linear range. These 7 calibrators were run in duplicate across 17 runs. There was no significant fixed or proportional bias based on Passing-Bablok regression slope and intercept with 95% confidence intervals which cross 1 and 0 respectively. There was a mean bias of 0.01 log_10_ fg/mL (95% confidence interval -0.1 to 0.11 log_10_ fg/mL).

**Supplemental Figure 2.** Respiratory swab cycle threshold (Ct) value compared to log_10_ nucleocapsid antigen concentration (fg/mL) in 74 plasma samples drawn within ±1 day of diagnostic respiratory reverse transcription quantitative polymerase chain reaction (RT-qPCR), analyzed by RT-qPCR test. The GeneXpert E gene and N2 gene panels (top left and top right respectively) show C_t_ values from the two targets in the same 21 samples. The remaining samples were tested by lab developed E gene test (n=30) and Panther Fusion targeting ORF1ab (n=23). Solid blue line represents the linear regression line, with shaded area representing the 95% confidence interval. The dashed horizontal line represents the plasma antigen positivity threshold.

**Supplemental Figure 3.** Comparison of plasma nucleocapsid antigen concentration by days from symptom onset in 777 plasma samples from 104 unique individuals with respiratory nucleic acid amplification test (NAAT)-confirmed, analyzed by outcome of death (A) vs. survival (B). Boxplots of the first positive sample from each unique individual each week after symptom onset show that antigen concentration is not associated with outcome of death at any time point (C). The dashed line represents the positivity threshold. Statistical significance from two-sided Wilcoxon rank sum testing is denoted as ns: *P* >.05, *: *P* ≤.05, **: *P* ≤.01, ***: *P* ≤.001.

**Supplemental Figure 4.** Comparison of plasma nucleocapsid antigen concentration by days from symptom onset in 777 plasma samples from 104 unique individuals with respiratory nucleic acid amplification test (NAAT)-confirmed COVID-19, subset by specimen type (A-C) and length of cold-storage time at 4°C (D-F). Boxplots of the first positive sample from each unique individual each week after symptom onset show that antigen concentration is not associated with either specimen type (C) or length of storage prior to processing (F) at any time point. The dashed line represents the positivity threshold. Statistical significance from two-sided Wilcoxon rank sum testing is denoted as ns: *P* >.05, *: *P* ≤.05, **: *P* ≤.01, ***: *P* ≤.001.

**Supplemental Figure 5.** Histogram (A), deviance residuals plot (B), and quantile-quantile plot (C) of the linear mixed-effects model of plasma nucleocapsid antigen concentration based on days from symptom onset and disease severity, accounting for inter-individual random variation, in 388 inpatient and ICU plasma samples collected between 5-40 days after symptom onset. They demonstrate an essentially normal distribution, except beyond the upper and lower 95^th^ percentiles, where observed values were more extreme than predicted. Deviance residuals appear approximately randomly distributed.

**Supplemental Figure 6.** See separate file.
