# Supplementary material for: SARS-CoV-2 Nucleocapsid Plasma Antigen for Diagnosis and Monitoring of COVID-19
Source: Clin Chem. 2021 Oct 4;68(1):204–13. doi: 10.1093/clinchem/hvab216 (PMC8522398; doi:10.1093/clinchem/hvab216)
Supplement: hvab216_Supplementary_Data [file hvab216_supplementary_data.zip › SuppFig6.pdf]

Supplemental Figure 6. Individual timeline plots for all 83 individuals with >1 plasma sample. They depict plasma nucleocapsid antigen concentration (top panel, green diamond, left side Y-axis), respiratory reverse transcription quantitative polymerase chain reaction (RT-qPCR) cycle threshold (C_t) values (top, orange + for positive RT-qPCR, orange x for negative RT-qPCR, right side inverted Y-axis), and anti-nucleocapsid antibody levels (bottom panel, red circle for IgA, blue square for IgG, purple triangle for IgM), by days from symptom onset. The dashed horizontal line represents threshold of positivity for both plasma antigen and respiratory RT-qPCR. The red dashed vertical line represents date of death.

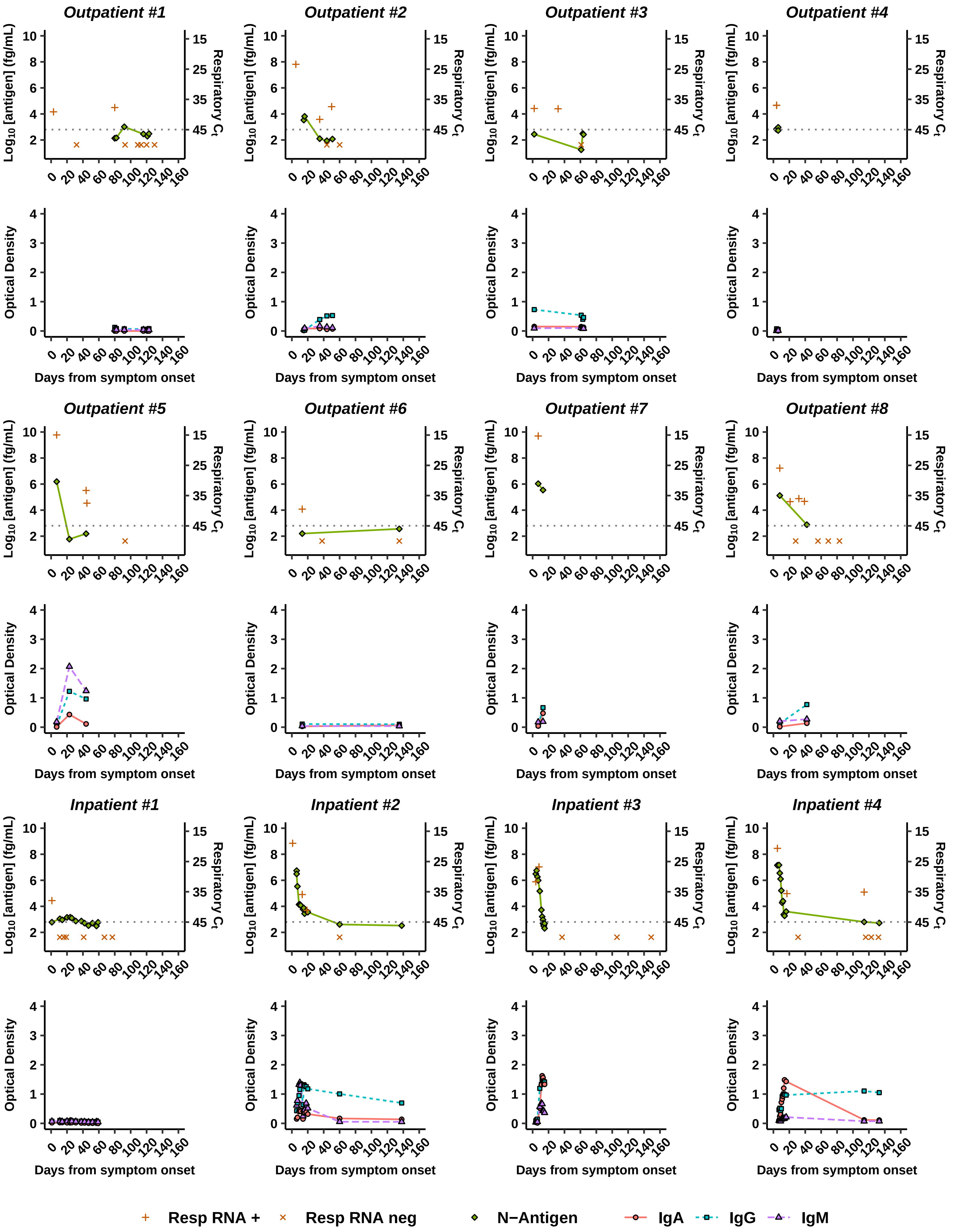

Outpatient #1
Outpatient #2
Outpatient #3
Outpatient #4
Outpatient #5
Outpatient #6
Outpatient #7
Outpatient #8
Inpatient #1
Inpatient #2
Inpatient #3
Inpatient #4
Log10 [antigen] (fg/mL)
Respiratory Ct
Optical Density
Days from symptom onset
Resp RNA +
Resp RNA neg
N−Antigen
IgA
IgG
IgM

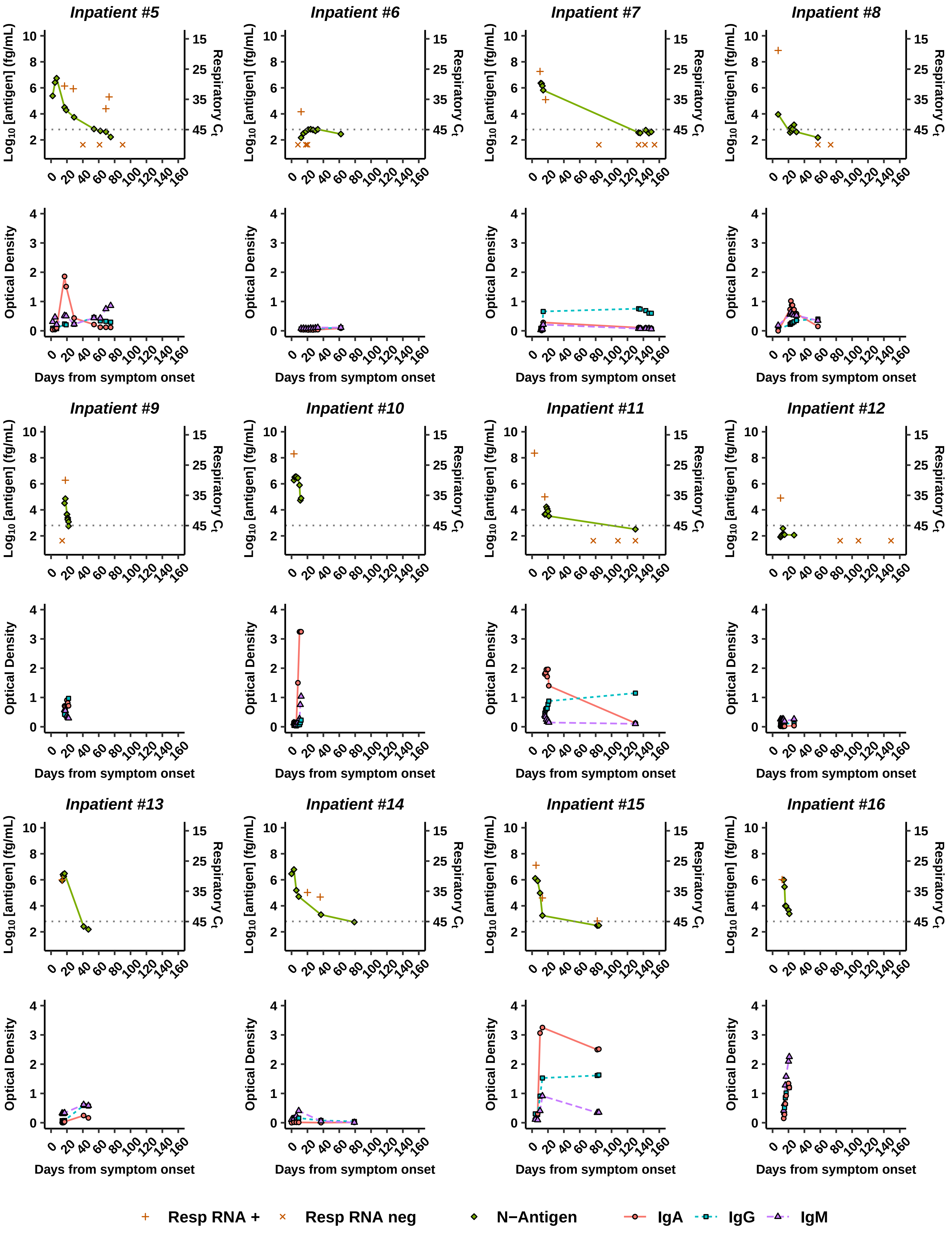

Inpatient #5
Inpatient #6
Inpatient #7
Inpatient #8
Inpatient #9
Inpatient #10
Inpatient #11
Inpatient #12
Inpatient #13
Inpatient #14
Inpatient #15
Inpatient #16
Log10 [antigen] (fg/mL)
Respiratory Ct
Optical Density
Days from symptom onset
Resp RNA +
Resp RNA neg
N−Antigen
IgA
IgG
IgM

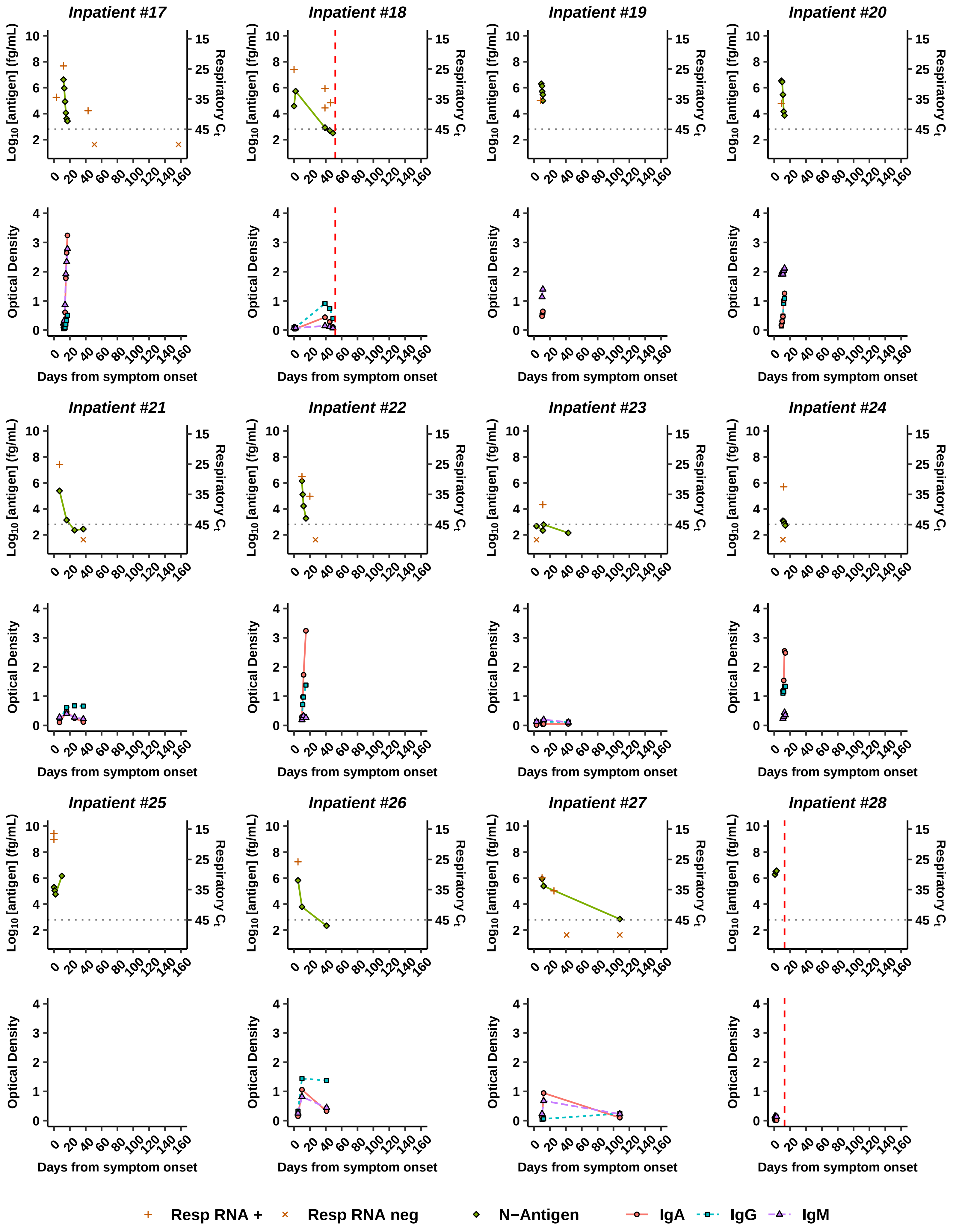

Inpatient #17
Inpatient #18
Inpatient #19
Inpatient #20
Inpatient #21
Inpatient #22
Inpatient #23
Inpatient #24
Inpatient #25
Inpatient #26
Inpatient #27
Inpatient #28
Log10 [antigen] (fg/mL)
Respiratory Ct
Optical Density
Days from symptom onset
Resp RNA +
Resp RNA neg
N−Antigen
IgA
IgG
IgM

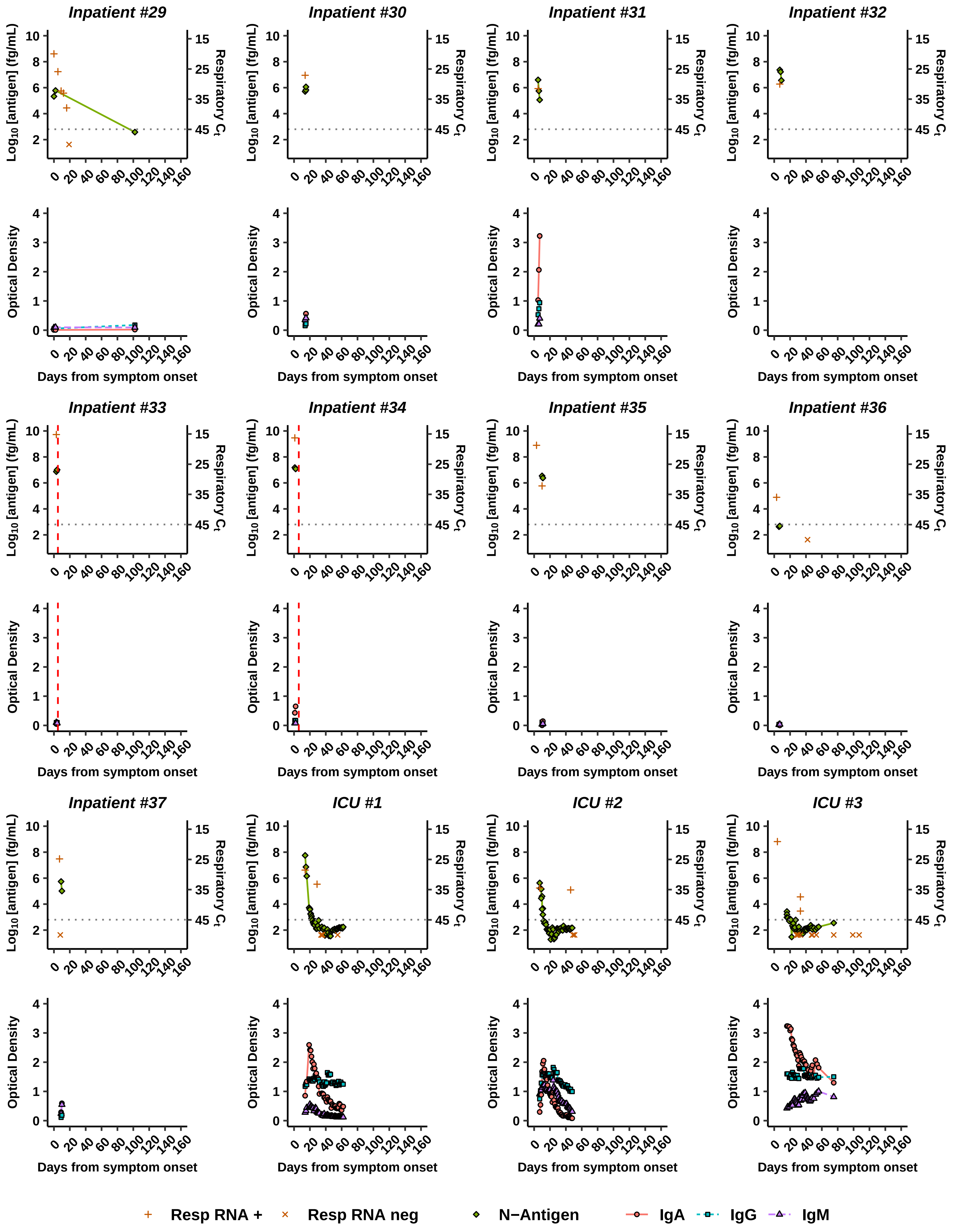
Inpatient #29
Inpatient #30
Inpatient #31
Inpatient #32
Inpatient #33
Inpatient #34
Inpatient #35
Inpatient #36
Inpatient #37
ICU #1
ICU #2
ICU #3
Log_{10} [antigen] (fg/mL)
Respiratory C_t
Optical Density
Days from symptom onset
Resp RNA +
Resp RNA neg
N−Antigen
IgA
IgG
IgM

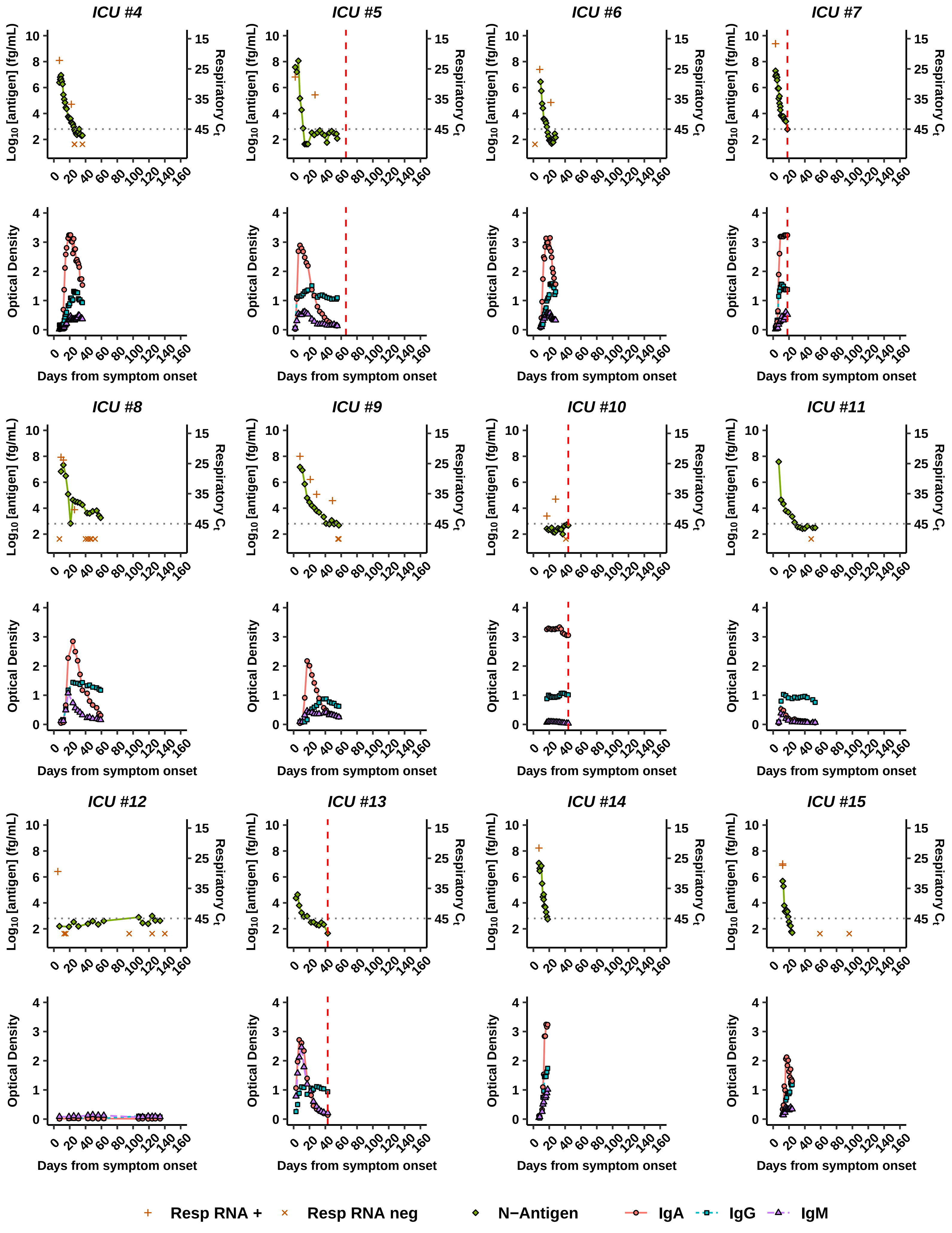
ICU #4
ICU #5
ICU #6
ICU #7
ICU #8
ICU #9
ICU #10
ICU #11
ICU #12
ICU #13
ICU #14
ICU #15
Log$_{10}$ [antigen] (fg/mL)
Respiratory C_t
Optical Density
Days from symptom onset
0 20 40 60 80 100 120 140 160
Resp RNA +
Resp RNA neg
N−Antigen
IgA
IgG
IgM

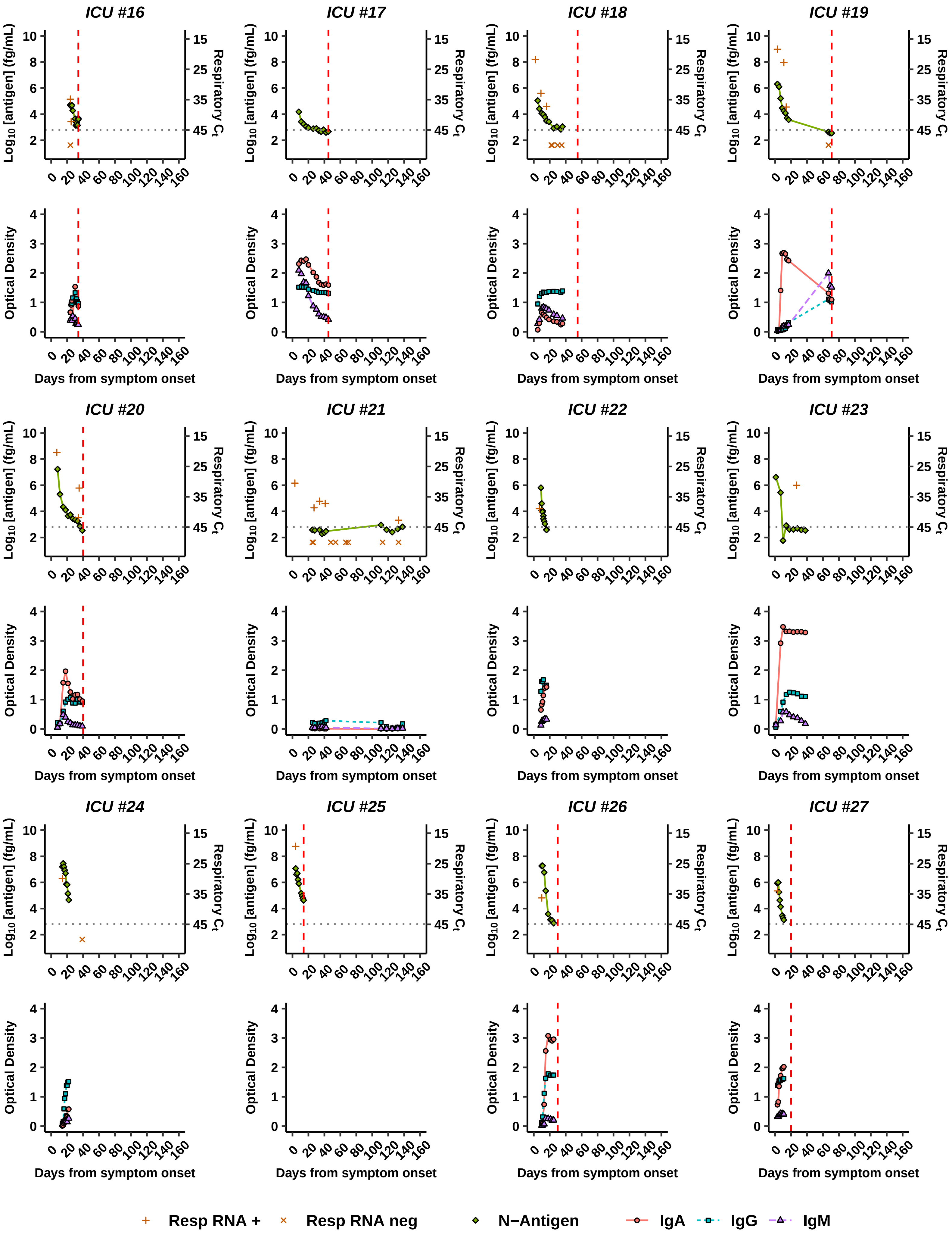

ICU #16
ICU #17
ICU #18
ICU #19
ICU #20
ICU #21
ICU #22
ICU #23
ICU #24
ICU #25
ICU #26
ICU #27
Log10 [antigen] (fg/mL)
Respiratory Ct
Optical Density
Days from symptom onset
Resp RNA +
Resp RNA neg
N−Antigen
IgA
IgG
IgM

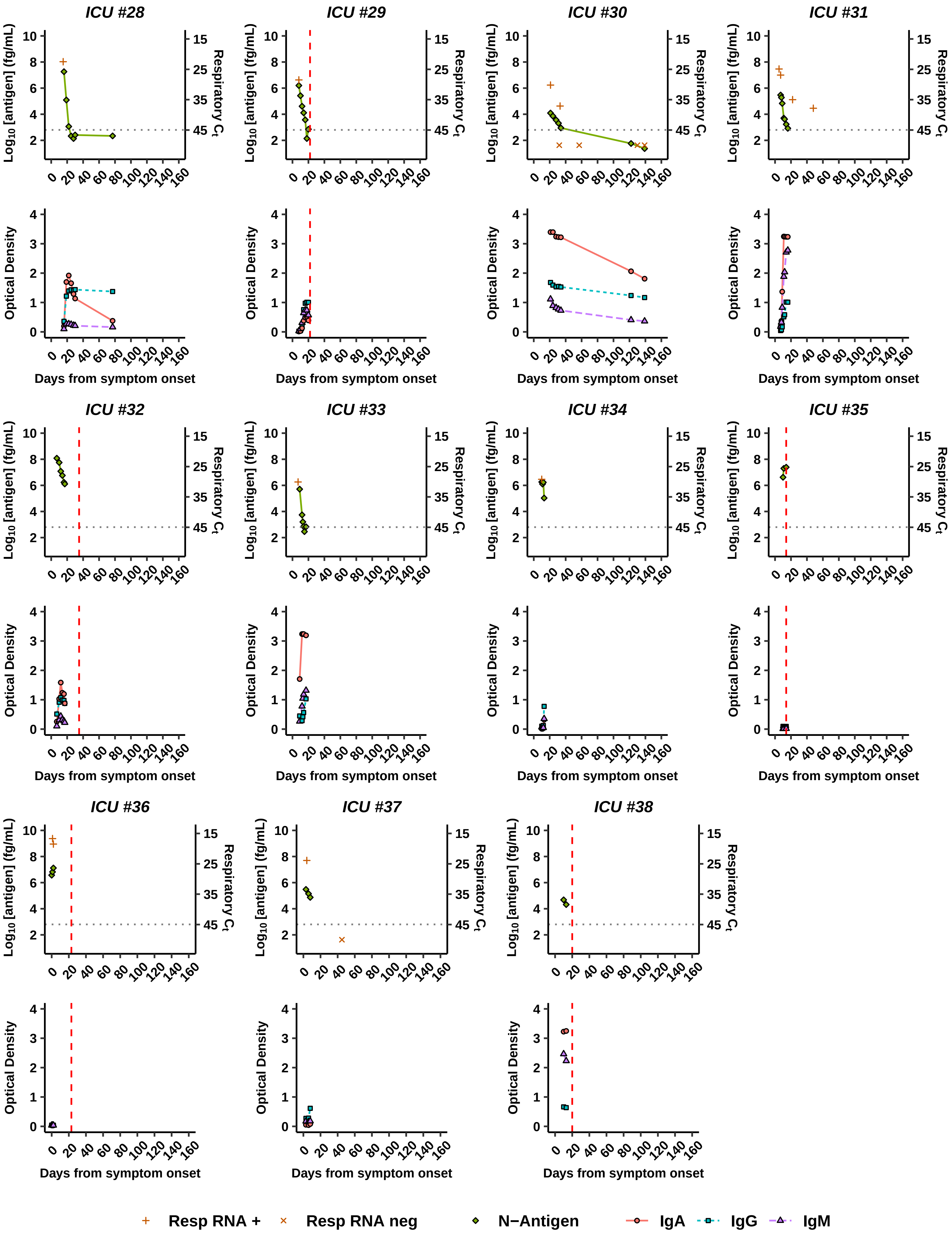
ICU #28
ICU #29
ICU #30
ICU #31
ICU #32
ICU #33
ICU #34
ICU #35
ICU #36
ICU #37
ICU #38
Log$_{10}$ [antigen] (fg/mL)
Respiratory C_t
Optical Density
Days from symptom onset
Resp RNA +
Resp RNA neg
N−Antigen
IgA
IgG
IgM
